# Supplementary material for: A Critical View of the Application of the APEX Software (Aqueous Photochemistry of Environmentally-Occurring Xenobiotics) to Predict Photoreaction Kinetics in Surface Freshwaters
Source: Molecules. 2019 Dec 18;25(1):9. doi: 10.3390/molecules25010009 (PMC7017383; doi:10.3390/molecules25010009)
Supplement: Supplementary file 1 [file molecules-25-00009-s001.zip › 3.2.4_gcc-4.4.0.zip!/3.2.4_gcc-4.4.0/3.2.4_gcc-4.4.0/mingw32/doc/drmingw/drmingw.html]

Dr. Mingw


Dr. Mingw is a *Just-in-Time (JIT)* debugger. When the application throws
an unhandled exception, Dr. Mingw attaches itself to the application and collects
information about the exception, using the available debugging information.

# Features

Dr. Mingw can read debugging information in *Stabs* formats — generated
by the Gnu C/C++ Compiler, and in a PDB file — generated by the Microsoft
Visual C++ Compiler.

Windows NT/2000 platform is supported, as well as Windows 95/98. On older Windows
versions though, the IMAGEHLP.DLL isn't included or it's a rather old version.
Dr. Mingw doesn't require it, but relies upon it to resolve symbols in modules
compiled by the Microsoft tools. See The
IMAGEHLP.DLL Saga for more information.

# Download

Dr. Mingw is now part of mingw-utils.

# Installation

If you didn't download the self-installing package, to install enter

> ```
> drmingw -i
> ```

Dr. Mingw will register itself as the JIT debugger by writting into the
system registry. On Windows NT/2000 make sure you have Administrator
rights. See Enabling Just-in-Time (JIT) Debugging for
more information.

If the installation is sucessful, the following message box should appear:

To enable other options they must be set them allong with the **-i**
option. For example,

> ```
> drmingw -i -v
> ```

If you still have trouble installing, edit the included **drmingw.reg** file to reflect the **drmingw.exe** executable path and
load it on the system registry.

# Usage

You can test Dr. Mingw by running the sample **test.exe**. Depending
of your Windows version, you'll see a familiar dialog:

If you request to debug the program, Dr. Mingw will attach to the
faulting application, collect information about the exception, and display
the dialog

To resolve the addresses it's necessary to compile the application with
debugging information. In case of address is in a DLL with no debugging
information, it will resolve to the precedent exported symbol.

# Command Line Options

The Dr. Mingw command line uses the following syntax:

```
drmingw [-h | --help] [-V | --version] [-i | --install] [-a | --auto] [-u | --uninstall]
[-p pid | --process-id=pid]  [-e event | --event=event]
[-v | --verbose]
```

The following table describes the Dr. Mingw command-line options. All
comand-line options are case-sensitive.

| Option |  | Action |
| --- | --- | --- |
| ``` -h ``` | ``` --help ``` | Print help and exit |
| ``` -V ``` | ``` --version ``` | Print version and exit |
| ``` -i ``` | ``` --install ``` | Install as the default JIT debugger |
| ``` -a ``` | ``` --auto ``` | Automatically start (used with **-i** \| **--install**) |
| ``` -u ``` | ``` --uninstall ``` | Uninstall |
| ``` -p pid ``` | ``` --process-id=pid ``` | Attach to the process with the given identifier |
| ``` -e event ``` | ``` --event=event ``` | Signal an event after process is attached |
| ``` -v ``` | ``` --verbose ``` | Verbose output |

# The EXCHNDL.DLL

## Introduction

Although internally Dr. Mingw behaves much like a debugger, from the
outside it is like a standalone exception handler. But for its own
debugging purposes, Dr. Mingw has a internal exception handler that is
completly seperate from the main code.

This exception handler resides in **exchndl.c**. When
**drmingw.exe** is loaded, the code in **exchndl.c** is automatically
executed (by the gcc static constructor/destructor mechanism) and registers
itself as a exception handler.

This exception handler is much lighter than Dr. Mingw debugging system
because it doesn't have to deal with interprocess communication. The
exception handling routine runs in the same process context of the faulting
application (**drmingw**, in this case).

## Using ExcHndl for you own purposes

If you incorporate ExcHndl in you own programs, especially those that
you release to others, you can have almost the same exception information
that you would get with Dr. Mingw, but with no need of the end user to have
Dr. Mingw installed.

You can use ExcHndl in two ways:

- linking **exchndl.o** and **libbfd.a** with your program objects
- linking **exchndl.o** and **libfd.a** in the
  **EXCHNDL.DLL** and dinamically loading it at run-time. This can be
  done by linking just **exchndl2.o** or explicitly calling
  *LoadLibrary("exchndl.dll")*

The latter method is preferred because you have smaller executables and
don't need to link the BFD library in all builds. The application wont fail
if the **EXCHNDL.DLL** is missing.

## Example

The sample **test.exe** application uses the second method above.
Copy **EXCHNDL.DLL** to executable directory. When you run it, even
before general protection fault dialog box appears, it's written to the
**test.RPT** file a report of the fault.

Here is how **test.RPT** should look like:

```
-------------------

Error occured on Sunday, May 7, 2000 at 20:22:03.

C:\home\jrfonseca\drmingw\src\test.exe caused an Access Violation in module C:\WINDOWS\system32\msvcrt.dll Writing to location 00000008.

Registers:
eax=00003039 ebx=00000064 ecx=00000008 edx=0244fec0 esi=00401211 edi=0244fec0
eip=78027470 esp=0244fcd8 ebp=0244fea8 iopl=0         nv up ei pl nz na pe nc
cs=001b  ss=0023  ds=0023  es=0023  fs=0038  gs=0000             efl=00010202

Call stack:
78027470  C:\WINDOWS\system32\msvcrt.dll:78027470  wscanf
7802544B  C:\WINDOWS\system32\msvcrt.dll:7802544B  sscanf
00401241  C:\home\jrfonseca\drmingw\src\test.exe:00401241  YetAnotherFunction  //C/home/jrfonseca/drmingw/src/test.cxx:14
00401272  C:\home\jrfonseca\drmingw\src\test.exe:00401272  MyWonderfulFunction  //C/home/jrfonseca/drmingw/src/test.cxx:19
004012A9  C:\home\jrfonseca\drmingw\src\test.exe:004012A9  main  //C/home/jrfonseca/drmingw/src/test.cxx:24
004011C1  C:\home\jrfonseca\drmingw\src\test.exe:004011C1
004011EB  C:\home\jrfonseca\drmingw\src\test.exe:004011EB
77E87903  C:\WINDOWS\system32\KERNEL32.dll:77E87903  SetUnhandledExceptionFilter
```

# Appendix

## Enabling Just-in-Time (JIT) Debugging

*[Taken from Using Microsoft Debuggers of the April 2000 Platform SDK.]*

There are a variety of application errors which will cause Windows
NT/Windows 2000 a to terminate the application. The most common kinds of
errors are deadlocks and access violations. From the operating systems
point of view, these are all simply unhandled exceptions.

When an application error occurs, Windows searches for an exception
handler. If it does not find an exception handler, the system verifies that
the application is not currently being debugged and considers the exception
to be unhandled. At this point, there are three possible responses:

- Windows can end the process immediately.
- Windows can freeze the process and start a user-mode debugger. This
  debugger can then be used to examine the application.
- Windows can run a debugging tool which will create a memory dump
  file of the application's memory space, and then end the process.

The debugging tool which is used to debug the application or write the
dump file is called *Just-in-Time (JIT) Debugger*, or the
*post-mortem debugger*.

The default JIT debugger is Dr. Watson. When the application throws
an unhandled exception, Dr. Watson attaches itself to the application and
generates a crash dump file. After it creates the crash dump file, Dr.
Watson closes the application and exits.

Any user-mode debugging tool can be selected as the JIT debugger:

- To change the JIT debugger to WinDbg, run **windbg -I**. When
  WinDbg is the JIT debugger, WinDbg will be activated whenever an
  application crashes. See WinDbg Command Line Options for details.
- To change the JIT debugger to NTSD, you must edit the registry.
  When NTSD is the JIT debugger, NTSD will be activated whenever an
  application crashes.
- To change the JIT debugger back to Dr. Watson, run **drwtsn32
  -i**. When Dr. Watson is the JIT debugger, a memory dump file will
  be written to disk if an application crashes. See Dr. Watson Command
  Line Options for details.

Only a system administrator can alter the JIT settings.

If a JIT debugger has been installed, you can deliberately break into
the debugger from a user-mode application by calling the **DebugBreak**
function.

### Editing the Registry

The Just-in-Time debugging settings are stored in the registry, under
**\\HKEY\_LOCAL\_MACHINE\Software\Microsoft\Windows NT\CurrentVersion\AeDebug\**.
The two relevant keys in this directory are **Debugger** and
**Auto**.

The **Debugger** key's value shows the name of the debugger specified
to analyze application errors. The **Auto** key is either zero or
one.

When an unhandled application error occurs, Windows checks to see if the
**Debugger** and **Auto** keys exist.

If the **Auto** key equals zero and the **Debugger** value
contains the name of a valid debugger (such as WinDbg or NTSD), the message
box will have two buttons: **OK** and **Cancel**. If the **OK**
button is pressed, the application is terminated. If the **Cancel**
button is pressed, the debugger specified in the **Debugger** key is
started.

If the **Auto** key equals zero, but the **Debugger** key value is
empty, the message box will have only an **OK** button and no debugger
will start.

If the **Auto** key equals one, no message box appears. The debugger
referred to in the **Debugger** key is automatically started.

**Setting Dr. Watson as the JIT debugger (default):**

```
Debugger = "drwtsn32 -p %ld -e %ld -g"
Auto = 1
```

**Setting WinDbg as the JIT debugger:**

```
Debugger = "WinDbg -p %ld -e %ld"
Auto = 1
```

**Setting NTSD as the JIT debugger:**

```
Debugger = "NTSD -p %ld -e %ld -g"
Auto = 1
```

In these examples, -**p %ld** specifies the process ID that NTSD
will debug, -**e %ld** provides the event that caused the
exception, and -**g** causes the debugger to skip the initial
breakpoint. (Dr. Watson ignores the -**g** option.)

## The IMAGEHLP.DLL Saga

*[Taken from several Bugslayer articles of MSJ.]*

The IMAGEHLP.DLL symbol engine first appeared in Windows NT� 4.0. The
beta Windows NT 5.0 SDK had new parts of IMAGEHLP.H that dealt with source
and line information. In the meantime, the November 1998 Platform SDK
showed and the IMAGEHLP.DLL that shipped with it supported the new source
and line handling. There are several different versions of IMAGEHLP.DLL.
The only one that does not support the new source and line information is
the original Windows NT 4.0 version.

The IMAGEHLP.DLL version 5.00.1678.1 dynamically links to MSPDB50.DLL.
It first tries to load MSDBI.DLL, and if that is not found it will load
MSPDB50.DLL, so it works with both Visual C++� 5.0 and 6.0. To use it with
Visual C++ 6.0, copy MSPDB50.DLL to MSPDB60.DLL. If you want to get symbols
from the field, you will have to compile with CodeView� symbols and use
.DBG files to get them. Keep in mind that MSPDB50.DLL, like MSDBI.DLL, is
not redistributable.

The IMAGEHLP.DLL version 5.00.1878.1, which comes with the Windows 2000
Beta 2 Platform SDK, hard links against MSDBI.DLL instead of dynamically
loading MSPDB50.DLL as in earlier versions to read PDB files. The problem
is that MSDBI.DLL is not redistributable.

IMAGEHLP.DLL now uses DEBUGHLP.DLL.

If you want IMAGEHLP.DLL, it's available in:

- Platform SDK
- WinDBG Debugger
- Windows 2000

# Suggested Reading

- A
  Crash Course on the Depths of Win32 Structured Exception Handling, MSJ
  January 1997
- MSJEXHND -
  Part 1, Under the Hood, MSJ April 1997
- MSJEXHND -
  Part 2, Under the Hood, MSJ May 1997
- Bugslayer,
  MSJ, August 1998
- The
  Win32 Debugging Application Programming Interface
